# Early Multi-organ Point-of-Care Ultrasound Evaluation of Respiratory Distress During SARS-CoV-2 Outbreak: Case Report

**DOI:** 10.5811/cpcem.2020.4.47524

**Published:** 2020-04-15

**Authors:** Robert Farrow, Graham Becherer-Bailey, Daniel Mantuani, Arun Nagdev

**Affiliations:** Highland Hospital / Alameda Health System, Department of Emergency Medicine, Oakland, California

**Keywords:** COVID-19, SARS-CoV-2, ultrasound, respiratory distress

## Abstract

**Introduction:**

Coronavirus disease 2019 (COVID-19) is caused by the virus known as severe acute respiratory syndrome coronavirus 2 (SARS-CoV-2). Several case series from Italy and China have highlighted the lung ultrasound findings of this disease process and may demonstrate its clinical utility during the current pandemic.

**Case Report:**

We present a case of a COVID-19 patient who presented to the emergency department twice within a 24-hour period with rapidly progressing illness. A multi-organ point-of-care ultrasound (POCUS) evaluation was used on the return visit and assisted clinical decision-making.

**Discussion:**

A multi-organ POCUS exam allows for quick assessment of acute dyspnea in the emergency department. As the lung involvement of COVID-19 is primarily a peripheral process it is readily identifiable via lung ultrasound. We believe that when applied efficiently and safely a POCUS exam can reduce clinical uncertainty and potentially limit the use of other imaging modalities when treating patients with COVID-19.

**Conclusion:**

This case highlights the utility of an early multiorgan point-of-care assessment for patients presenting with moderate respiratory distress during the severe SARS-CoV-2 pandemic.

## INTRODUCTION

Point-of-care ultrasound (POCUS) examinations of patients with acute respiratory distress have been demonstrated to be useful for patients with acute unexplained dyspnea in the emergency department (ED).^1^ Multiple previous ED studies have demonstrated the ability of clinicians to rapidly and accurately differentiate a cardiac etiology (specifically acute decompensated congestive heart failure) versus other causes of acute dyspnea.^1^,^2^ In our early experience during the severe acute respiratory syndrome coronavirus 2 (SARS-CoV-2) outbreak, with multiple patients presenting with acute dyspnea of suspected parenchymal pulmonary pathology, we found that the prompt differentiation between an underlying cardiac versus pulmonary source can be instrumental in both triage and early resuscitation.

Early reports from China detailed the utility of computed tomography (CT) in demonstrating the classic multifocal, ground-glass opacities that are commonly present in patients with pulmonary manifestations of the rapidly progressing viral pandemic.^3^ Our early, multi-organ ultrasound first strategy in the evaluation of the severely dyspneic patient centers on not assuming all patients who arrive in our ED during this large wave of patients are purely pulmonary in nature (even though we recognize the high prevalence of disease), and using a multi-organ POCUS examination to help guide initial treatment and resuscitation. We also believe that incorporating a system-based approach in this manner will allow a reduction in CT imaging and reduce the ever-present issues of departmental contamination and CT disinfection.

Recent studies out of China have detailed ultrasound findings associated with coronavirus disease 2019 (COVID-19).^4^,^5^ While these findings are not specific, they are likely clinically useful in patients with severe dyspnea in conjunction with a multi-organ evaluation of cardiac function and the inferior vena cava (IVC). Furthermore, we believe that these non-specific ultrasound findings can be used in conjunction with clinical and laboratory parameters to assist defining pulmonary involvement of SARS-CoV-2, especially as it typically involves peripheral lesions near the pleura, which are well demonstrated on lung ultrasound. Herein we present a case of SARS-CoV-2 related multifocal pneumonia diagnosed by POCUS in the ED during the initial triage of a return ED visit, which highlights its clinical utility and our proposed imaging pathway for evaluating patients with acute dyspnea during the current SARS-CoV-2 outbreak.

## CASE REPORT

A 56-year-old female with a past medical history of asthma and dyslipidemia presented to a community ED with one week of fever, non-productive cough, dyspnea, headache, nausea and vomiting. She denied smoking history or drug use. Travel history was significant for returning home from an amusement park in Los Angeles one week prior to onset of symptoms. Vital signs at triage were temperature (oral) 38.6° Celsius; heart rate 117 beats per minute; respiratory rate 20 breaths per minute; and pulse oximetry 93% on room air.

### Clinical Course on First Emergency Department Visit

**Image 1:**
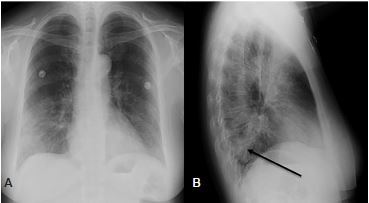
Chest radiographs of the initial emergency department visit. Patient was dyspneic at the time. A) Posterior-anterior view demonstrating prominent interstitial markings in the mid and lower lung field bilaterally; and B) Lateral view with apparent area of retrocardiac opacity (arrow) likely representing an early consolidation in the left lateral lobe.

Significant laboratory results were as follows: rapid influenza diagnostic test was negative; white blood cells 11.7 10*3 per microliter (mcL) (reference range 4.5–11.5 10*3/mcL), neutrophils relative 92% (reference 50–70%), lymphocyte absolute 0.55 10*3/mcL (reference 0.8–4.80 10*3 /mcL), and lactic acid was 1.0 millimoles per L (reference 0.5–2.2 mmol). A two-view chest radiograph (CXR) was interpreted by the radiologist as pneumonia of the left lower lobe with interstitial changes (Image 1). After symptomatic therapy and a first dose of azithromycin, the patient was discharged home with instructions to continue antibiotic therapy and return for worsening symptoms.

**CPC-EM Capsule**

What do we already know about this clinical entity?

The virus severe acute respiratory syndrome coronavirus 2 can cause severe pulmonary infection and inflammatory response in patients presenting during the coronavirus disease 2019 (COVID-19) pandemic.

What makes this presentation of disease reportable?

This case highlights the rapid progression of COVID-19 pneumonia and the utility of point-of-care-ultrasound (POCUS) in excluding alternative causes of dyspnea.

What is the major learning point?

Multiorgan-POCUS is useful for ED evaluation of dyspnea during the COVID-19 pandemic due to the peripheral nature of lung involvement.

How might this improve emergency medicine practice?

Multiorgan-POCUS has the potential to reduce diagnostic uncertainty in dyspneic patients and help limit use of other imaging modalities.

### Clinical Course Second Emergency Department Visit

Approximately 12 hours after discharge the patient returned to the same ED with worsening dyspnea. Upon arrival she was noted to be ill appearing, tachypneic and with moderate respiratory distress despite similar triage vital signs as the initial ED visit. Her lung exam was significant for poor inspiratory effort and rhonchi at the bases. A multi-organ POCUS exam was performed to determine the cause of the patient’s dyspnea.

**Image 2:**
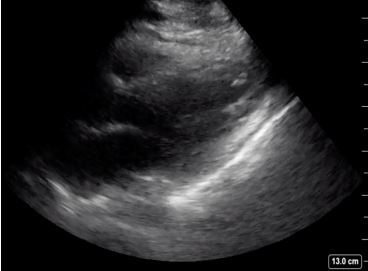
Normal systolic ejection fraction determined by parasternal long-axis view. Image acquisition via Sonosite X-Porte system using phased array probe.

**Image 3:**
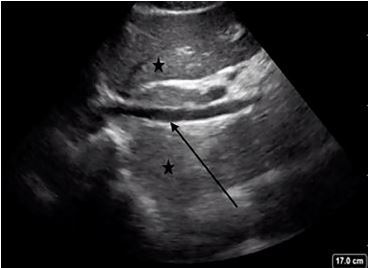
The inferior vena cava (arrow) was determined to be non-plethoric and collapsible with respiration. In this image, the inferior vena cava is surrounded by the liver (stars). Image acquisition via Sonosite X-Porte system using phased array probe.

**Image 4:**
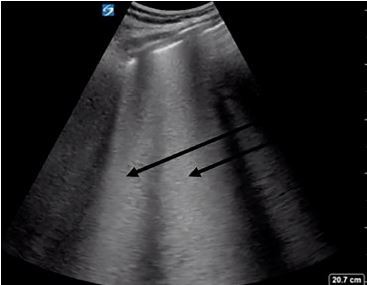
Confluent B-lines were seen in all lung fields with increased density in the posterior lateral sections. The arrows denote B-lines that were interpreted as pulmonary infiltrates due to depth and confluence. Image acquisition via Sonosite X-Porte system using curvilinear probe.

A cardiac parasternal long-axis view demonstrated normal systolic ejection fraction and no pericardial effusion (Image 2). The IVC in the subxiphoid view showed greater than 50% collapse during inspiration (Image 3). A lung exam using a low frequency (5–2 megahertz) curvilinear transducer in the anterior, lateral, and posterior portions, showed the presence of diffuse scattered B-lines with small subpleural consolidations and effusions in each lung zone, with confluent B-lines in the posterior inferior lobes bilaterally (Image 4). POCUS findings were interpreted as the presence of a non-cardiogenic multifocal interstitial lung process with COVID-19 being high on the differential. The patient was placed in a negative pressure room, and all staff were informed to wear full personal protective equipment when interacting with the patient.

**Image 5:**
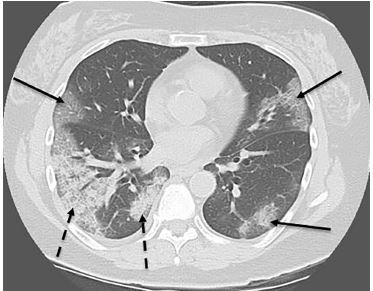
Computed tomography of the chest without contrast in an axial cut showing diffuse multifocal infiltrates (solid arrows) with areas of consolidation and increased infiltrates in the posterior segments (dashed arrows).

Based on the POCUS multi-organ findings, the patient was resuscitated with an intravenous bolus normal saline and treated with broad-spectrum antibiotics (levofloxacin). SARS-CoV-2 oropharyngeal and nasopharyngeal swabs were sent from the ED for testing. The infectious disease consultant agreed with a plan for admission and continuation of droplet, airborne, and contact precautions given the progression of symptoms and worsening respiratory status. She was admitted to a negative pressure room on the medical floor for continued therapy and monitoring of respiratory status. An eventual non-contrast CT of the chest demonstrated diffuse multifocal infiltrates (Image 5).

As an inpatient, the patient’s antibiotic regimen was adjusted to doxycycline and cefepime. She was under observation and isolation for six days at the hospital of presentation. At this point the SARS-CoV-2 testing had not yet resulted, but her clinical course had improved. She was discharged home with instructions to self-quarantine at home or return to the ED if symptoms worsened. SARS-CoV-2 testing resulted the day after discharge as positive for SARS-CoV-2 ribonucleic acid.

## DISCUSSION

During the current SARS-CoV-2 pandemic, prompt evaluation of patients in the ED presenting with acute dyspnea is imperative. Diagnostic testing of SARS-CoV-2 has been limited to date, and in our setting will not result during a typical ED visit. Likewise, serum laboratory markers for both SARS-CoV-2 associated pneumonia and non-SARS-CoV-2 causes of acute dyspnea (decompensated heart failure, chronic obstructive pulmonary disease/asthma, pulmonary embolism) are non-specific, are also not immediately resulted, and are of limited value during the initial hospital presentation.

The role of imaging during the SARS-CoV-2 outbreak is still being established. A study of patients diagnosed with COVID-19 in Wuhan, China, demonstrated a progression of disease by CT imaging from early subclinical/asymptomatic patients with unilateral and multifocal ground-glass opacities to patients with less than one week of symptoms showing bilateral disease and transition to consolidation and interstitial changes.^6^ However, the American College of Radiology recently issued guidance that CT should not be used as a first-line test to diagnose acute SARS-CoV-2 infection, and that limiting the use of portable radiography should be attempted to reduce transmission.^7^

POCUS holds some distinct advantages over other imaging modalities especially in times of disaster or pandemic. The characteristic ultrasonographic findings of interstitial pneumonia near the pleura are accessible, rapidly attained and reliable markers of pathology. Lung ultrasound has been shown to be more sensitive than CXR for pneumonia and pulmonary edema.^8^,^9^ In our anecdotal experience, ultrasonographic features of COVID-19 may be detectable earlier or more reliably than on CXR. Additionally, while assessing for findings of interstitial pneumonia, basic cardiac and IVC imaging is easily obtainable and can offer information in regard to the presence of an alternative pathology and guide resuscitation.

While the majority of patients infected with SARS-CoV-2 will experience only mild illness, a subset will progress to multifocal pneumonia, acute respiratory distress syndrome, and cardiomyopathy^10^–^12^ pathologies that can be identified rapidly with POCUS.^8^,^9^,^13^–^15^

## CONCLUSION

The above case highlights the utility of a multiorgan approach in the evaluation of the acutely dyspneic patient during the SARS-CoV-2 pandemic. Along with lung ultrasound findings that have been described in China and Italy, we believe that the evaluation of the heart and IVC are easily obtained and extremely useful for two important reasons. First, this approach allows to rapidly determine other common causes of dyspnea in the undifferentiated patient. Second, with a more protocolized pathway on early presentation, we hope to reduce the reliance on other imaging modalities (chest radiographs and CT) in a time when infection control is imperative. In our experience, a multi-organ ultrasound first approach for all severely dyspneic patients is an ideal approach during the global SARS-CoV-2 pandemic and especially as healthcare resources become increasingly strained.
